# Progress in Natural Compounds/siRNA Co-delivery Employing Nanovehicles for Cancer Therapy

**DOI:** 10.1021/acscombsci.0c00099

**Published:** 2020-10-23

**Authors:** Milad Ashrafizadeh, Ali Zarrabi, Kiavash Hushmandi, Farid Hashemi, Ebrahim Rahmani Moghadam, Mehdi Raei, Mahshad Kalantari, Shima Tavakol, Reza Mohammadinejad, Masoud Najafi, Franklin R. Tay, Pooyan Makvandi

**Affiliations:** † Faculty of Engineering and Natural Sciences, Sabanci University, Orta Mahalle, Üniversite Caddesi No. 27, Orhanlı, Tuzla, 34956 Istanbul, Turkey; ‡ Sabanci University Nanotechnology Research and Application Center (SUNUM), Tuzla 34956, Istanbul Turkey; § Department of Food Hygiene and Quality Control, Division of Epidemiology & Zoonoses, Faculty of Veterinary Medicine, University of Tehran, Tehran 1419963114, Iran; ∥ Department of Comparative Biosciences, Faculty of Veterinary Medicine, University of Tehran, Tehran, Iran; ⊥ Department of Anatomical Sciences, School of Medicine, Student Research Committee, Shiraz University of Medical Sciences, Shiraz 7134814336, Iran; # Health Research Center, Life Style Institute, Baqiyatallah University of Medical Sciences, Tehran 1435916471, Iran; ¶ Department of Genetics, Tehran Medical Sciences Branch, Azad University, Tehran 19168931813, Iran; □ Cellular and Molecular Research Center, Iran University of Medical Sciences, Tehran 1449614525, Iran; ■ Pharmaceutics Research Center, Institute of Neuropharmacology, Kerman University of Medical Sciences, Kerman 7616911319, Iran; ○ Medical Technology Research Center, Institute of Health Technology, Kermanshah University of Medical Sciences, Kermanshah 6715847141, Iran; ● Radiology and Nuclear Medicine Department, School of Paramedical Sciences, Kermanshah University of Medical Sciences, Kermanshah 6715847141, Iran; △ College of Graduate Studies, Augusta University, Augusta, Georgia 30912, United States; ▽ Istituto Italiano di Tecnologia, Centre for Micro-BioRobotics, viale Rinaldo Piaggio 34, 56025 Pontedera, Pisa Italy; ▲ Department of Medical Nanotechnology, Faculty of Advanced Technologies in Medicine, Iran University of Medical Sciences, 14496-14535 Tehran, Iran

**Keywords:** anticancer therapy, chemotherapy, co-delivery platforms, nanocarriers, natural products, small interfering RNA

## Abstract

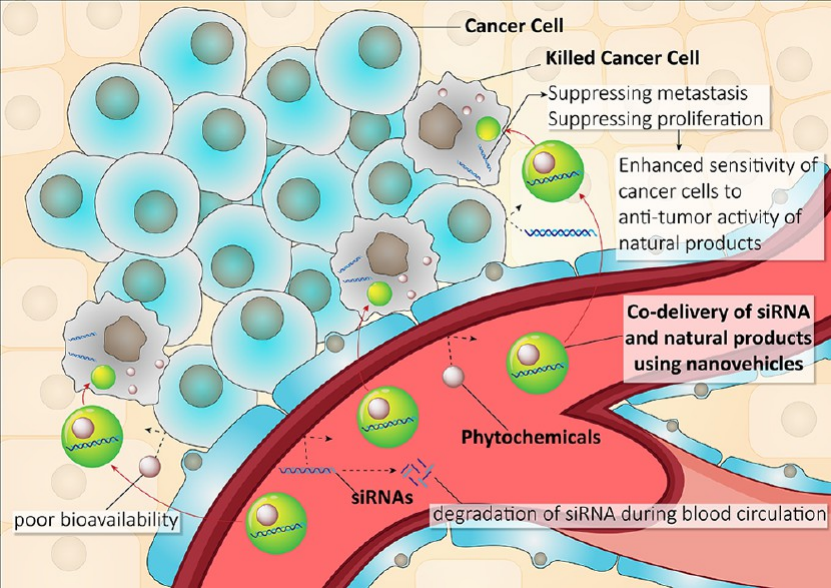

Chemotherapy using natural compounds, such as resveratrol, curcumin, paclitaxel, docetaxel, etoposide, doxorubicin, and camptothecin, is of importance in cancer therapy because of the outstanding therapeutic activity and multitargeting capability of these compounds. However, poor solubility and bioavailability of natural compounds have limited their efficacy in cancer therapy. To circumvent this hurdle, nanocarriers have been designed to improve the antitumor activity of the aforementioned compounds. Nevertheless, cancer treatment is still a challenge, demanding novel strategies. It is well-known that a combination of natural products and gene therapy is advantageous over monotherapy. Delivery of multiple therapeutic agents/small interfering RNA (siRNA) as a potent gene-editing tool in cancer therapy can maximize the synergistic effects against tumor cells. In the present review, co-delivery of natural compounds/siRNA using nanovehicles are highlighted to provide a backdrop for future research.

## Introduction

According to the World Health Organization (WHO), 9.6 million deaths are attributed to cancer. This life-threatening disorder was the second leading cause of death worldwide in 2018.^1^ Despite considerable progress in anticancer therapy, many challenges still exist.^2,3^ One of the challenges is the off-targeting feature of conventional cancer therapeutics that significantly diminishes their therapeutic efficacy.^4,5^

In light of this, research scientists have focused on using targeted delivery in overcoming cancer cells. Notably, targeted delivery systems are able to inhibit tumor growth and reduce tumor burden.^6^ It is held that designing novel nanoscale delivery systems for delivery of siRNA can improve its efficacy in gene silencing. It appears that resistance of cancer cells to chemotherapy has limited the potential of targeted delivery systems. SiRNA is a powerful tool in reversing chemoresistance of cancer cells by down-regulation of oncogene factors, such as Survivin, Bcl-xl, and Mcl-1.^7,8^ Thus, understanding the mechanisms involved in drug resistance can help render anticancer therapy more efficacious.^9^ Another issue in anticancer therapy is the low efficacy of monotherapy in the eradication of cancer cells.^10^ These difficulties have spurred scientists toward developing co-delivery strategies for anticancer therapy. Combination cancer treatment indeed has significant appeal owing to its many advantages over monodelivery therapeutics, including improved efficacy by synergistic effects and overcoming drug resistance.^11−13^ In this regard, various siRNA and natural compounds co-delivery vehicles have been developed to achieve more effective therapy than conventional monodelivery.^14^ Natural compounds, because of their biobased origin, have attracted more attention than synthetic drugs.^15^ The present Review aims to provide a summary of the potential of natural compounds-siRNA co-delivery platforms in the elimination of cancer cells and suppression of their resistance to chemotherapy.

## Natural Compounds in Anticancer Therapy: An Overview

Natural compounds have opened new vistas in anticancer therapy because of their structural and chemical diversity.^15−18^ These compounds are of importance in the field of drug discovery that can lead to the discovery of novel cancer therapeutics.^19−21^ More than 100 natural products and their analogs are currently applied clinically or in clinical trials.^22,23^ Between 1981 and 2010, up to 50% of antitumor drugs approved by the US Food and Drug Administration (FDA) are natural compounds or their analogs.^24^ Accordingly, natural products are important in anticancer therapy. Numerous experiments have evaluated the efficacy of natural products in anticancer therapy. Because of their multitargeting capability, natural compounds can negatively affect the different aspects of cancer cells, such as proliferation, viability, and metastasis.^25−32^ In this way, natural compounds target various molecular pathways. The most common manner in which natural products participate in anticancer therapy is stimulation of apoptotic cell death.^33^ Administration of natural products induces mitochondrial-mediated and endoplasmic reticulum (ER)-mediated apoptosis.^34,35^ Natural products enhance the production of reactive oxygen species (ROS) that stimulate mitochondrial dysfunction, as well as ER stress.^36,37^ By increasing ROS generation, the integrity of the mitochondrial membrane is disrupted. During this process, expression of the antiapoptotic factor Bcl-2 is down-regulated,^38^ while the pro-apoptotic factor Bax is up-regulated. This causes the release of cytochrome C (Cyt C) from the mitochondria and activation of the caspase cascade that results in apoptosis.^39^ Another pathway is the induction of ER stress-mediated apoptosis.^40^ Natural product supplements trigger ER stress by enhancing ROS generation. This, in turn, causes apoptotic cell death by upregulation of C/EBP homologous protein (CHOP).^41^ In addition to apoptotic cell death, natural products are capable of targeting molecular pathways involved in the proliferation of cancer cells. The PI3K/Akt signaling pathway is a vital axis for the proliferation and growth of cancer cells.^42^ This pathway can be inhibited by an onco-suppressor factor known as PTEN.^43^ Studies have demonstrated that natural products are capable of activating PTEN in suppressing the PI3K/Akt signaling pathway, thereby decreasing the proliferation and viability of cancerous cells.^44^ Manu natural products that can target molecular pathways involved in metastasis and invasion of cancer cells.

Epithelial-to-mesenchymal transition (EMT) is a process that causes metastasis of cancer cells via malignant transformation of epithelial cells into mesenchymal cells.^45,46^ Natural products have shown potential in suppressing EMT to minimize their migration and improve cancer prognosis.^47^ The upstream modulators of EMT can also be targeted by natural products. It is held that Wnt and STAT3 are upstream modulators of EMT in cancer.^48,49^ The administration of natural products inhibits both Wnt and STAT3 to suprress EMT.^50,51^ In addition, ZEB proteins that induce EMT during cancer metastasis are also down-regulated by natural products.^52^

Natural products are promising candidates in anticancer therapy due to their capacity in affecting diverse targets such as growth and migration of cancer cells as well as targeting different molecular pathways.^53−55^ However, the poor bioavailability of these valuable compounds has negative impact on their anticancer therapeutic activity.^56^ The application of nanocarriers can remarkably enhance the antitumor potential of natural products, protect them against degradation before reaching the tumor sites, and augment their accumulation in cancer cells via penetrating into the blood-tumor barrier (BTB).^56−59^ These benefits support the use of nanoparticles for natural product delivery in anticancer therapy.

## SiRNA: Basics, Role in Anticancer Therapy, Challenges, and Possible Strategies

Conventional therapeutics have drawbacks, of which the limitation in targeting just one special molecular pathway or protein is the most important.^60,61^ Consequently, attention has been directed toward using genetic tools in anticancer therapy.^62^ RNA interference (RNAi) is one of the most powerful genetic tools used in anticancer therapy.^63^ Cancer occurs as a result of mutations in onco-suppressor and oncogene factors, leading to uncontrolled cell growth and inhibition of apoptosis.^64,65^ Different driver genes accounting for enhancing growth and malignancy of cancer have been identified.^66^ RNA interference is beneficial in the modulation of the aforementioned genes in anticancer therapy.^67,68^ The discovery of RNAi and its application have a long history; RNAi was first discovered in plants. Subsequently, scientists attempted to exploit the potential of RNAi in gene editing. In 2006, Fire and Mello received the Nobel prize in medicine because of their significant contribution in the field of RNAi.^69^ The extensive application of RNAi in anticancer therapy is not accidental. The high specificity, effectiveness, minimal adverse effects, and ease of preparation of RNAi has led to its use in anticancer therapy.^70^

Small interfering RNA (siRNA) is a subcategory of small RNA molecules with a length of 21–23 nucleotides.^71^ To adequately performing its function, siRNA requires a complete match with its target mRNA (mRNA).^72^ Furthermore, siRNA suppresses the expression of its target gene at the post-transcriptional level by mRNA degradation.^73^ Biogenesis of siRNA commences via the degradation of long double-stranded RNA in the cytoplasm via Dicer enzyme. For activation, siRNA is embedded into an RNA-induced silencing complex (RISC) to produce single-stranded RNA (ssRNA). This ssRNA functions as an antisense guide for the RISC complex. By binding to a complementary mRNA target, the ssRNA causes degradation via Argonaute proteins.^74,75^

Application of first synthetic siRNA dated back to 2001 when Elbashir and colleagues used siRNA for gene editing in mammalian cells.^76^ Other scientists followed by using siRNA for gene silencing in anticancer therapy.^77,78^ Because of the capability of siRNA in selective targeting, much attention has been directed toward using siRNA in treatment of different cancers, Examples include breast cancer,^79^ lung cancer,^80^ brain tumors,^81^ thyroid cancer,^82^ and bladder cancer.^83^ Recent publications have shed some light on using siRNA in anticancer therapy. Oncogene factors participating in cancer malignancy may be targeted via SiRNA. The remodeling and spacing factor-1 (RSF-1) is an oncogene factor that is high expressed in cancer cells. Up-regulation of RSF-1 enhances the proliferation of cancer cells and causes resistance of cancer cells to chemotherapy.^84^ The siRNA-mediated RSF-1 silencing in cervical cancer cells is associated with their enhanced sensitivity to radiotherapy. Down-regulation of RSF-1 by siRNA increases the efficacy of radiotherapy via stimulation of apoptosis, DNA damage, and cell cycle arrest in cervical cancer cells.^85^ Apart from RSF-1, glucose transporter-1 (GLUT-1) is also responsible for resistance of cancer cells to radiotherapy;^86,87^ siRNA-induced GLUT1 inhibition render cancer cells more responsive to radiotherapy by induce their DNA damage and apoptosis.^88^ These two studies illustrate that siRNA is a potential strategy in enhancing the efficacy of radiotherapy. Invasion and metastasis of cancer cells may be regulated with the use of siRNA. Matrix metalloproteinase-2 (MMP-2) is a proteinase that enhances the migration of cancer cells and promotes lymph node metastasis via the degradation of type IV basement membrane collagen.^89^ The siRNA-mediated Annexin A7 inhibition reduces proliferation and invasion of cancer cells via down-regulation of MMP-2 and *proliferating cell nuclear antigen* (PCNA).^90^ Ribonucleotide reductase (RR) is a potential target in anticancer therapy because of its role in DNA repair and replication via catalytic reduction.^91^ Ribonucleotide reductase regulatory subunit M2 (RRM2), a protein-coding gene, is expressed during the late G1/early S phase and participates in DNA repair.^92^ RRM2 induces chemoresistance of cancer cells because of its capabililty in DNA repair.^93^ In ovarian cancer cells, silencing of RRM2 via siRNA induces DNA damage and inhibits their repair. This, in turn, increases the sensitivity of cancer cells to cisplatin chemotherapy.^94^

The signaling networks responsible for proliferation, metastasis, radioresistance and chemoresistance of cancer cells have been reported in previous studies. Targeting molecular pathways is important in suppressing the aggressive behavior of cancer cells and in promoting their responses to chemotherapy and radiotherapy. However, siRNA suffers from off-targeting and are easily degraded by enzymes. These drawbacks may be circumvented by using nanosized vehicles. Similar to the encapsulation of natural product cargoes, encapsulation of siRNA by nanocarriers protect them against degradation during blood circulation. Nanomaterials can also provide targeted delivery of siRNA to the tumor site. Potential nanocarriers for delivery of siRNA in anticancer therapy will be reviewed in the next section.

**Figure 1:**
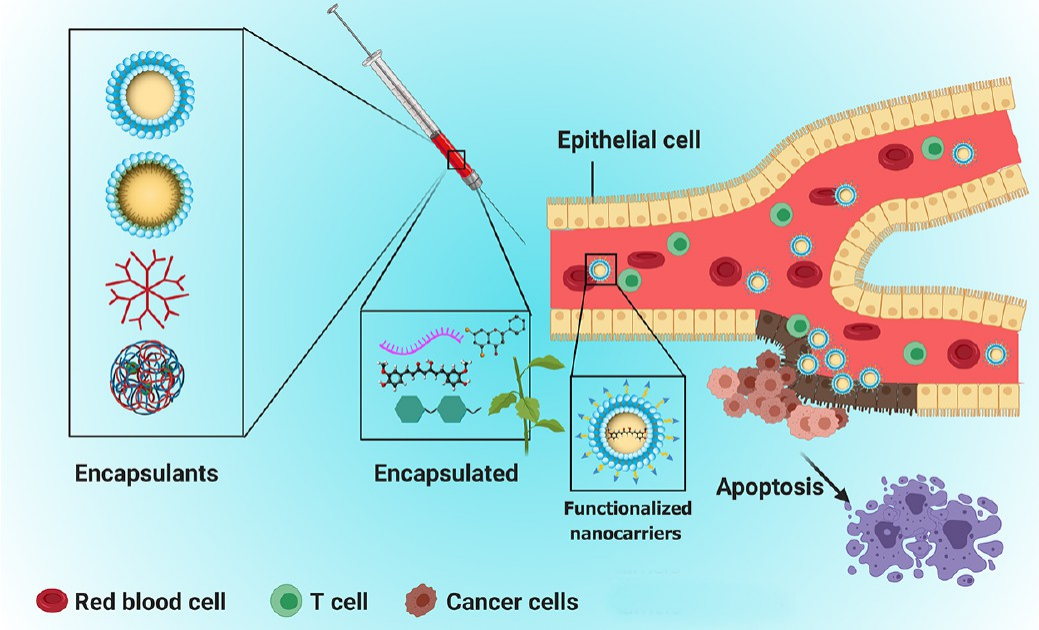
Anticancer therapy using a site-specific co-delivery strategy. SiRNA and phytochemicals can be coloaded on nanoparticles for promoting their efficacy in cancer therapy. Encapsulation of siRNA in nanoparticles protects against degradation. Nanoparticles enhance bioavailability of natural products. Blood circulation time of siRNA and phytochemicals increases by nanoparticles. Various nanoparticles, such as micelles, liposomes, dendrimers, and polymeric nanoparticles can provide targeted delivery of siRNA and phytochemicals at tumor site, leading to an increase in their efficacy in apoptosis induction.

Because different therapeutics employed for combination cancer treatment have specific sites and mechanisms of action, nanovehicle-mediated co-delivery strategies are essential for maximizing the synergistic effects against tumor cells.^14^ In light of this, functionalized vehicles with site specific delivery have attracted substantial attention in precisely delivering multiple therapeutic agents/RNA for improved synergistic effects (Figure 1).

Although siRNAs are important in anticancer therapy, there are a number of extracellular and intracellular barriers that challenge their efficacy.^71^ Among these siRNA limitations, off-targeting, their instability in blood circulation, inadvertent stimulation of the host’s immune responses, as well as their incapability to enter cells (cell uptake) are the most important.^95^ With respect to off-targeting, it has been reported that one-tenth of siRNAs affect unintended genes.^78^ In addition, siRNAs triggers immunotoxicity by inducing inflammation and enhancing the levels of cytokines.^96^ Synthetic siRNAs may impair RNAi machinery by interfering with the function of microRNAs (miRs) and stimulating the overexpression of specific proteins.^97^ The most critical challenge of siRNAs is their hydrophilic and anionic features that inhibit their penetration through hydrophobic cellular membranes.^98^

To circumvent this issue, various delivery platforms have been developed for siRNAs. To date, polymeric nanoparticles, gold nanoparticles, iron oxide nanoparticles, silicon dioxide nanoparticles, carbon nanotubes, lipid nanoparticles, liposomal nanoparticles, hydrogel nanoparticles, and aptamers have been developed for delivery of siRNAs.^99^ Recent literatures have reported the usefulness of siRNA-delivery systems in anticancer therapy. Dendrimers are a subcategory of polymeric nanoparticles with three components, including a central core, an internaldendritic structure and an external surface with the functional surface group. Dendrimers are promising candidates for the delivery of anticancer drugs.^100^ SiRNA can be loaded into dendrimers for anticancer therapy. Dendrimers remarkably enhance the cellular uptake of siRNAs and their release from endosomes. This causes more effective up-regulation or down-regulation of their targets, resulting in decrease in cancer malignancy.^101^ Selenium nanoparticles are beneficial in drug and gene delivery. These nanoparticles overcome multidrug resistance (MDR) because of their great biocompatibility and high cellular uptake.^102,103^ Selenium nanoparticles not only reduce adverse effects, they also enable maximum gene silencing.^104^ Because of their low size (<100 nm), nanoparticles can infiltrate cellular impediments, such as the blood–tumor barrier (BTB), the blood–brain barrier (BBB), and the cell membrane.^105,106^ It has been reported that siRNA-loaded nanocarriers can penetrate BBB via endocytosis and transcytosis,^107^ resulting in more effective treatment of brain tumors. Reduction in off-targeting and adverse effects, enhancement of therapeutic capability and elevation of cellular uptake are the benefits of using nanoparticles for siRNA delivery.^108−111^Table 1 summarizes the different nanocarriers used for siRNA delivery in anticancer therapy.

**Table 1:** siRNA-Loaded Nanoparticles in Anticancer Therapy

nanovehicle	cancer type	cell line	target gene	size (nm)	zeta potential (mV)	encapsulation efficiency (EE) (%)	drug	results	ref
polymeric nanoparticles	pancreatic cancer	HEK293T cell line	GRP78	92	+15.14	27–31		high efficiency in silencing GRP78 gene (83.9% decrease in expression) and cytotoxicity against cancer cells	(112)
lipid/polymer hybrid nanoassembles	prostate cancer	PC3 cells	EGFR	120.2	–8.8	98		reducing growth and volume of cancer without making toxicity against normal cells	(113)
lipid nanoparticle	ovarian cancer	human ovarian cancer SK-OV-3 cells	RPN2	66.5	–9.1	more than 80		effective gene silencing, and excellent cellular uptake	(114)
multifunctional nanoplatform	lung cancer	human lung adenocarcinoma A549 cells	PLK1	80–102	5–12	78–80		providing endo/lysosomal escape, having a pH-responsive feature to release a drug in the tumor microenvironment, high cellular uptake, and cytotoxicity	(113)
redox-responsive nanoparticles	liver cancer	human hepatic (L02) and hepatoma cells (HepG2)	Bcl-2	85		80	camptothecin	accumulation and selective targeting of cancer cells, and induction of apoptosis via Bcl-2 down-regulation	(115)
silica nanoparticles	breast cancer	human breast carcinoma cell line MDA-MB-231	PLK1	100–200	–19			effective elimination of cancer cells via down-regulation of PLK1	(116)
magnetic nanoparticles	prostate cancer	PC3 cell line	ADAM10	15.82–79.20	5–31			high cellular uptake and reducing expression of ADAM10, leading to a decrease in cell viability	(115)
polymeric nanoparticles	liver cancer	Huh7 cells	survivin	210	–6.7	53		stimulation of apoptosis in cancer cells via down-regulation of surviving and subsequent induction of Bax and caspase-3	(117)
selenium nanoparticles	cervical cancer	HeLa human cervical cancer cell	derlin-1	less than 150	14.7			enhancing generation of ROS, stimulation of mitochondrial dysfunction and induction of apoptotic cell death	(118)
magnetic nanoparticles	oral cancer	human oral cancer cell Ca9–22 and CAL 27	Bcl-2	26.12	46.5			decreasing viability and survival of cancer cells via down-regulation of Bcl-2	(119)
pH-responsive micelles	liver cancer	human liver cancer cells SK-Hep1	IL-8	83				high biocompatibility, excellent cellular uptake and effective decrease in gene expression	(120)

## Natural Compounds–siRNA Co-delivery

### Doxorubicin–siRNA Co-delivery

Doxorubicin (DOX) belongs to the family of anthracyclines and is extensively employed for the treatment of breast cancer, lung cancer, ovarian cancer, cervical cancer, and thyroid cancer.^121^ Doxorubicin is derived from bacteria belonging to the genus *Streptomyces*. It suppresses malignancy and proliferation of cancer cells via inhibition of DNA topoisomerases, DNA intercalation, and free radical generation.^122^ Despite its excellent antitumor activity, DOX adversely affects normal cells because of its off-targeting feature.^123,124^ This has resulted in using nanoplatforms for the targeted delivery of DOX.^125^ In addition, cancer cells are capable of developing resistance against DOX chemotherapy.^126^ These two issues have resulted in the use of combination therapy and nanoparticles. It has been shown that siRNAs are helpful in reversing DOX chemoresistance by targeting the genes involved in DOX resistance.

A combination of DOX and siRNA has been used for enhancing the antitumor activity of DOX against cancer cells. Chemotherapeutic agents can reduce the malignancy of cancer cells via EMT induction.^127^ Different molecular pathways function as an upstream regulators of EMT in cancer. The Ras-related C3 botulinum toxin substrate 1 (RAC1) is considered as a key player in the regulation of invasion and metastasis of cancer cells.^128,129^ The RAC1 attaches to nicotinamide adenine dinucleotide phosphate (NADPH) oxidase (NOX) and increases the production of ROS.^130^ Formation of actin stress fibers subsequently occurs by cytoskeleton reorganization.^131^ Down-regulation of RAC1 suppresses metastasis of cancer cells via inhibition of EMT. The use of DOX and siRNA-RAC1 enhances the antitumor activity of DOX against breast cancer cells via inhibition of EMT.^132^ The antitumor effect of DOX is augmented by elevating its accumulation in cancer cells by inhibition of P-gp activity via siRNA.^133^ The use of siRNA enables negative targeting of oncogene factors, such as STAT3, β-catenin, and Notch-1, which increases the antitumor activity of DOX.^134^ Molecular pathways involved in proliferation and growth of cancer cells, such as PI3K/Akt, may be targeted using siRNA, resulting in an increase in cytotoxicity of DOX against cancer cells.^135^ These studies are in support of the value of collaborative antitumor therapy via DOX and siRNA.^136−138^ Previous studies have examined the potential of co-delivery of siRNA and DOX using nanoparticles in anticancer therapy.^139^

The advent of nanotechnology facilitates simultaneous chemotherapy and immunotherapy. Programmed death-ligand 1 (PD-L1) is the key element of the PD-1/PD-L1 axis that induces apoptosis of T cells, inhibits their proliferation and provides immune escape of cancer cells.^140,141^ Down-regulation of PD-L1 is a potential strategy in the elimination of cancer cells by enhancing the cytotoxicity of T cells against tumor cells.^142^ The combination of DOX and siRNA-PD-L1 is beneficial in anticancer therapy. Cancer cell membrane-coated nanoparticles (CCMNPs) are capable of codelivering DOX and siRNA–PD-L1. Improved cellular uptake of CCMNPs enhances the internalization of PD-L1 and DOX, resulting in concomitant chemotherapy and immunotherapy.^143^ Internalization of DOX in cancer cells may be improved by targeting transporters. The role of P-gp in exporting chemotherapeutic agents out of the cell has previously been reported.^144^ Loading siRNA–MDR1 on nanoparticles for co-delivery with DOX is important for enhancing the antitumor activity of DOX. Expression and activity of P-gp are reduced by down-regulation of MDR1. This results in increased accumulation of DOX in cancer cells to improve its antitumor activity.^145^

Surface modification of nanoparticles with receptors and ligands can be made to enhance their targeted delivery. The EphA10 demonstrates high expression in cancers and is correlated with the progression and malignancy of cancer cells.^146^ Surface modification of nanoparticles with EphA10–antibody enhances their cellular uptake, leading to effective inhibition of P-gp and cytotoxicity of DOX.^147^ Following the design of nanoparticles that are capable of increasing intracellular DOX uptake, the next step should be devoted to developing strategies in reducing the viability and proliferation of cancer cells to maximize the antitumor activity of DOX. In this way, siRNA–Bcl-2- and DOX-loaded liposomes have been designed. By down-regulation of the antiapototic factor Bcl-2, the cancer cells undergo apoptosis and increase their sensitivity to DOX-mediated cell death.^148^ Nanoparticles are valuable for targeted delivery and enhanced cellular uptake of siRNA–Bcl-2 and DOX in anticancer therapy.^149^

**Figure 2:**
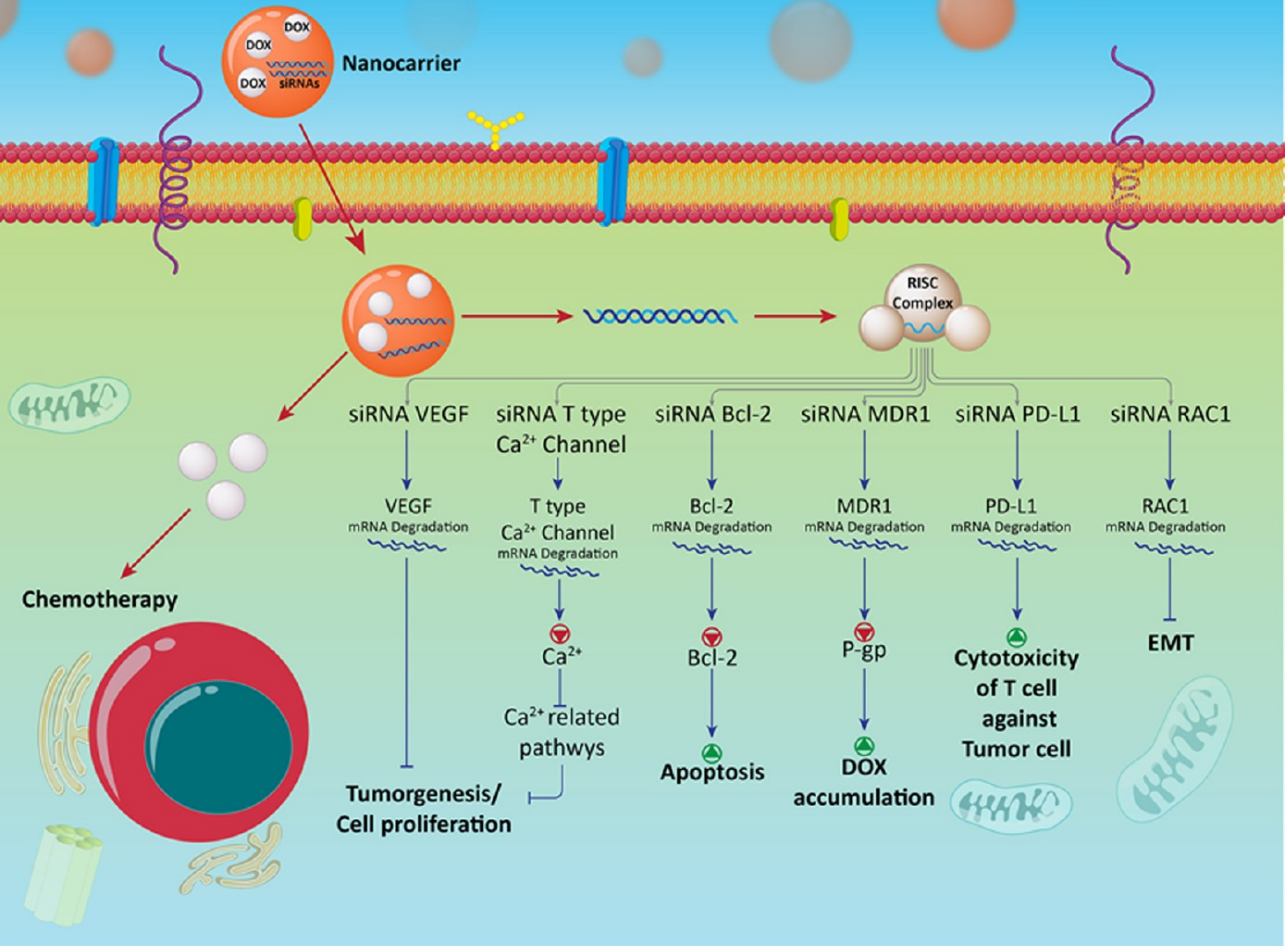
Co-delivery of DOX–siRNA in anticancer therapy and affected molecular pathways. Nanovehicles facilitate the penetration of siRNA and DOX through the cell membrane. SiRNA down-regulates molecular pathways that are responsible for cancer progression to promote antitumor activity of DOX.

Cytosolic Ca^2+^ is a vital signal transduction regulator that has a variety of biological functions, such as modulation of cell proliferation, tumorigenesis, and migration.^150−152^ The Ca^2+^ channels and pumps accounting for Ca^2+^ transportation are up-regulated in different cancers.^153−155^ These pumps increase the concentration of Ca^2+^ in the cells to activate Ca^2+^-related pathways.^156^ Activation of Ca^2+^-related pathways induces drug resistance.^157^ As a consequence, attention has been directed toward inhibition of Ca^2+^ pumps, such as low-voltage activated T-type Ca^2+^ channels in anticancer therapy.^158−160^ Encapsulation of siRNA against T-type Ca^2+^ channels and DOX by mesoporous silica nanoparticles reduces the activity of these channels, resulting in inhibition of DOX resistance in breast cancer cells.^161^ In addition to siRNA, other plant derived-natural compounds may be loaded into nanoparticles. The co-delivery of siRNA, quercetin and DOX suppresses proliferation and malignancy of cancer cells by providing collaborative antitumor therapy (Figure 2).^162,163^

The JNK-interacting protein 1 (JIP1) is an oncogene factor involved in the development of resistance against DOX by cancer cells. Down-regulation of JIP1 enhances the sensitivity of DOX chemotherapy.^164^ Co-delivery of JIP1 and DOX by cationic nanoliposomes inhibits the resistance of osteosarcoma cells to chemotherapy via induction of apoptosis and cytotoxicity.^165^ The erythropoietin-producing human hepatocellular *receptor A2* (EphA2) undergoes up-regulation in osteosarcoma cells. Loading of the histidine-tagged EphA2 receptor-specific peptide (YSA peptide) as a ligand of EphA2 into cationic nanoliposomes enhances the efficacy of delivery of siRNA and DOX into cancer cells.^165^ In addition to liposomes, graphene oxide may be used for DOX delivery. Graphene oxide is an oxidative product of graphite. The excellent biocompatibility and biodegradability of graphene oxide have made it valuable for drug delivery.^166−171^ Co-delivery of siRNA–VEGF and DOX using graphene oxide enhances their cellular uptake and targeted delivery, resulting in suppressing growth and metastasis of cancer cells.^172^

Apart from side effects, chemoresistance is a major problem associated with DOX-related chemotherapy. Enhanced metastasis is correlated with DOX resistance. EMT inhibition via siRNA leads to DOX sensitivity. Furthermore, P-gp that contributes to pumping out DOX from cancer cells and triggering chemoresistance is inhibited by siRNA.

Encapsulants offer a platform for co-delivery of DOX and siRNA to promote siRNA efficiency in gene silencing, and to increase DOX accumulation in cancer cells. The advantage of using siRNA is simultaneous chemotherapy and immunotherapy. For instance, siRNA-PD-L1 can be applied for preventing immune evasion of cancer cells to support the use of DOX in chemotherapy. siRNA-Bcl-2 may be used to promote the efficacy of DOX in apoptosis induction. To increase the selective targeting capability of nanocarriers, surface modification of nanoparticles with receptors, such as EphA10 has been adapted to promote their cellular uptake. Apart from DOX and siRNA, other antitumor agents, such as quercetin, can be loaded into nanoparticles to increase their efficacy against cancer cells. However, one of the drawbacks is the large particle size of nanoparticles. As shown in Table 2, most of synthesized NPs have particle size that are more than 100 nm. Future studies have to be focused on reducing the particle size of nanocarriers to enhance cellular uptake. Table 2 summarizes the DOX–siRNA co-delivery platforms used experimentally in anticancer therapy.

**Table 2:** DOX-siRNA Co-delivery Platforms in Anticancer Therapy^a^

nanovehicle	cancer type	cell line	target gene	size (nm)	zeta potential (mV)	encapsulation efficiency (EE) (%)	results	ref
ROS-sensitive NPs	breast cancer	4T1 cells	PD-L1	139.9	28.1		down-regulation of PD-L1, and providing simultaneous chemotherapy and immunotherapy	(173)
polymeric NPs	breast cancer	human breast cancer MCF-7 and MCF-7/ADR cell lines	P-gp	74.7	13.6		enhancing intracellular accumulation in cancer cells via down-regulation of P-gp	(174)
polymeric NPs	liver cancer	HepG2 cells	Bcl-2	60–90	less than 25	79.4	induction of apoptosis via down-regulation of Bcl-2	(175)
mesoporous silica NPs	oral cancer	human oral squamous carcinoma DOX-resistant cell line (KBV)	MDR1	170.5	+34.7		70% decrease in expression of MDR1, enhanced accumulation of DOX in cancer cells and stimulation of apoptosis	(176)
selenium NPs	liver cancer	HCC (HepG2) and human normal liver cell (Lo2)	Nanog	12			cellular uptake via clathrin-mediated endocytosis, down-regulation of Nanog, and inhibition of proliferation and migration	(177)
micelle	lung cancer	A549 cells	TLR4	125.9	+24.66	85.81 (DOX)	releasing drug and siRNA in a pH/redox-sensitive manner, and suppressing tumor growth	(178)
polymeric NPs	breast cancer	MCF-7 cells	MDR1	65.7	+13.9	67.4 (DOX)	inhibition of drug resistance via down-regulation of P-gp, and enhancing the antitumor activity of DOX	(179)
self-assembled polyjuglanin NPs	lung cancer	human lung cancer cell lines, A549 and H69	Kras	81.8	–18.62		down-regulation of oncogene factor Kras, inhibition of c-Myc and P-gp, and enhanced cytotoxicity of DOX	(180)
gold NPs	ovarian cancer	SK-OV-3 cells	erbB2	105	–48		targeted delivery, high biodistribution, and great antitumor activity	(181)
mesoporous silica NPs	breast cancer	human breast adenocarcinoma cell line MCF-7	Bcl-2	125	–47.4		targeted delivery and inhibition of cancer proliferation	(182)
gold NPs	cervical cancer	HeLa cells	EGFP	150	–35.4	82.5	inhibition of EGFP expression, high intracellular accumulation and suppressing cancer malignancy	(183)
polymeric NPs	breast cancer	MCF-7 cells	Bcl-2	187	+22.5		induction of apoptotic cell death via down-regulation of Bcl-2	(184)
chitosan NPs	lung cancer	A549 cells	IGF-1R	176	+11	86 (siRNA)75 (DOX)	suppressing invasion and migration of cancer cells via down-regulation of MMP-9, VEGF, and STAT3	(185)
micelles	breast cancer	4T1 and WRL-68 cells	MDR	92–101	+7 to +10	72 (DOX)	inhibition of resistance via down-regulation of MDR	(186)
micelles	breast cancer	MCF-7 cells	PLK-1	98.74	+21.62 to +44.5		suppressing proliferation of cancer cells	(187)
chitosan NPs	breast cancer	MDA-MB361 metastatic breast cancer cell line	IL17RB	114	+10.1		enhancing cytotoxicity of DOX via down-regulation of IL17RB, and inhibition of NF-κB and Bcl-2	(188)

a NP: Nanoparticles.

### Curcumin–siRNA Co-delivery

Curcumin is a naturally occurring nutraceutical compound derived from *Curcuma longa*.^189^ This compound is responsible for the yellow color of turmeric and is responsible for the purported therapeutic activities of *Curcuma longa*.^190,191^ Curcumin has a number of pharmacological effects such as neuroprotective,^192^ cardioprotective,^193^ hepatoprotective,^194^ antitumor,^195,196^ antioxidant,^197^ and anti-inflammatory effects.^198^ In terms of antitumor activity, many studies have reported the efficacy of curcumin in suppressing the proliferation, viability, and migration of cancer cells via targeting molecular pathways and mechanisms, such as apoptosis, autophagy, STAT3, Bcl-2, Bax, caspase, Wnt, and Nrf2.^199−203^ Similar to other plant-derived natural compounds, curcumin suffers from poor bioavailability.^204^ Loading curcumin into nanoparticles has been reported to remarkably enhance its antitumor activity.^205^ Curcumin has been used with gene therapy to augment its antitumor activity.^206,207^ Because of curcumin’s poor bioavailabililty, studies have focused on developing nanosized encapsulants for co-delivery of curcumin and siRNAs. To date, four studies have evaluated curcumin–siRNA co-delivery in anticancer therapy, which are summarized below.

**Figure 3:**
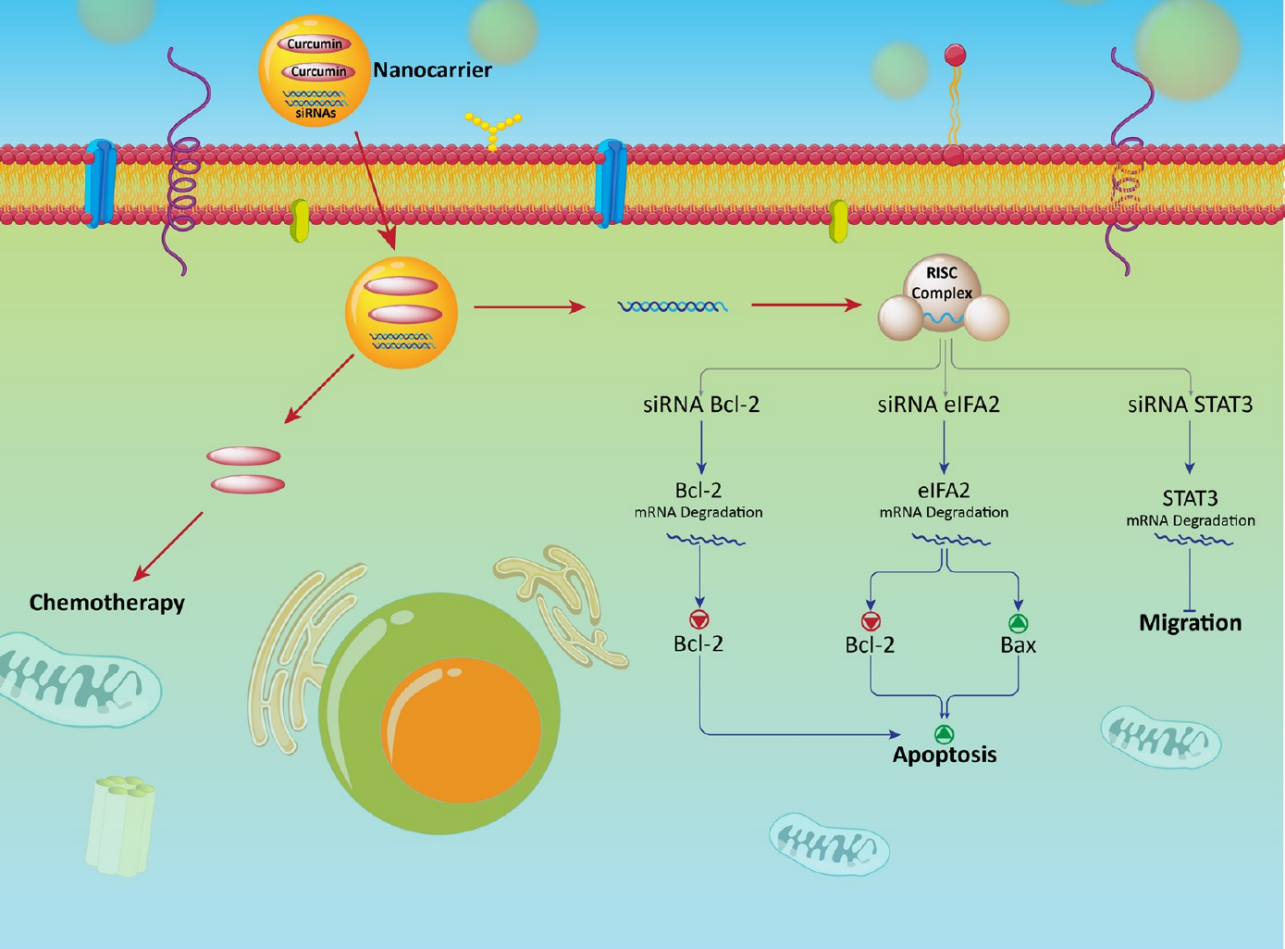
Co-delivery of curcumin and siRNA in cancer therapy with focus on molecular signaling pathways. Down-regulation of Bcl-2, elF5A2, and STAT3 by siRNA increases the antitumor activity of curcumin against cancer cells. Nanoparticles promote cellular accumulation of siRNA and curcumin to enhance their antitumor potential.

Polyamidoamine (PAMAM) dendrimers are promising candidates in drug and gene delivery because of the high density of surface groups, capability of sustained cargo release, spherical shape, low polydispersity, and water solubility.^208,209^ The hydrophobic interior of PAMAM dendrimers is ideal for the encapsulation of hydrophobic compounds, while their hydrophilic surface provides sites for attachment of siRNA.^210^ Both siRNA and curcumin can be codelivered by PAMAM dendrimers into cancer cells. Anticancerous effect was achieved by synergistic inhibition of Bcl-2 expression by the siRNA and antitumor acivity of curcumin (Figure 3).^211^

The STAT3 signaling pathway is an oncogene factor that enhances the proliferation and invasion of cancer cells.^212,213^ Down-regulation of STAT3 causes apoptosis of skin cancer cells and inhibits their migration and growth.^214,215^ Because curcumin targets the STAT3 signaling pathway in anticancer therapy, co-delivery of curcumin and STAT3-targeting siRNA can provide synergistic effects. In vitro and in vivo experiments demonstrate that curcumin- and siRNA–STAT3-loaded cationic liposomes are capable of suppress skin cancer progression and malignancy via down-regulation of STAT3 and disruption of cancer growth.^216^ The efficacy of cationic liposomes in the co-delivery of curcumin and siRNA-STAT3 in therapy against skin cancer was also investigated in another study. This combination remarkably suppressed skin cancer proliferation, growth, and survival.^217^ Because STAT3 in an oncogene for skin cancer (melanoma), silencing of STAT3 using siRNA interferes with cancer growth and invasion.

**Figure 4:**
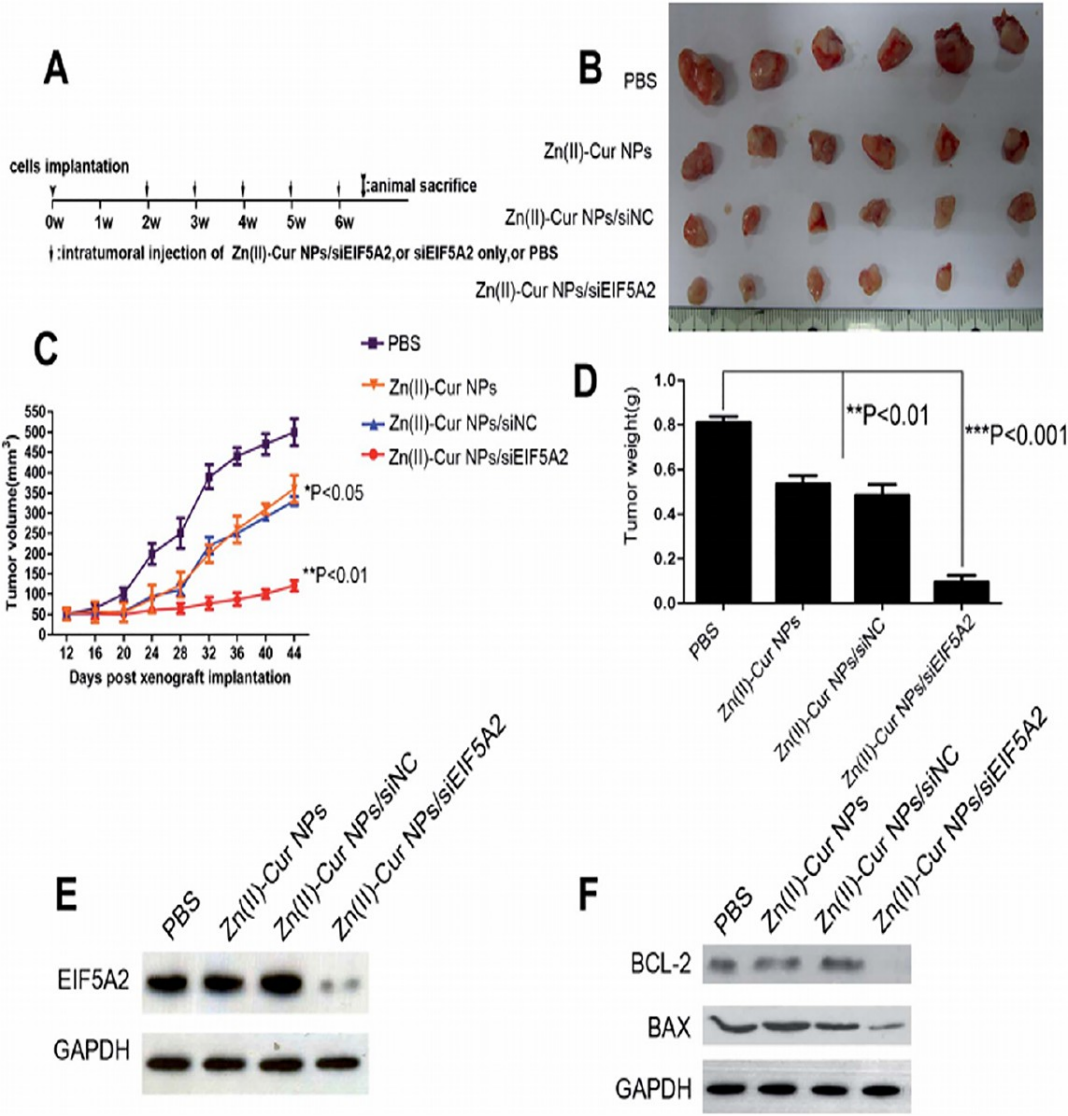
(A) Evaluation of antitumor potential of Zn(II)–Cur NP/siELF5A2 complex in a xenograft model. (B) Size of tumor treated with different therapeutics is shown. (C) Tumor volume based on days post xenograft implantation with Zn(II)–Cur NPs, Zn(II)–Cur NPs/siNC, and Zn(II)–Cur NPs/siEIF5A2 (20 mg of siEIF5A2 per injection, 50:1 mass ratio). (D) Mean tumor weights implanted with Zn(II)–Cur NPs, Zn(II)–Cur NPs/siNC, and Zn(II)–Cur NPs/siEIF5A2. (E) Western blots of specimens using anti-EIF5A2 and anti-GAPDH antibodies. (F) Western blots of the tissue specimens using anti-BCL-2, anti-BAX, and anti-GAPDH antibodies. Reproduced from ref (222) with permission from Royal Society of Chemistry.

Delivery of curcumin also enhances the inhibitory impact of STAT3 on melanoma cells. Nonviral vehicles, such as Au nanoparticles, carbon nanotubes, and silica nanoparticles are not biodegradable.^218−220^ Degradation of biodegradable polymers, such as poly(lactic-*co*-glycolic acid) nanoparticles, results in the production of acidic oligomers and creation of a low pH environment that are toxic for cells.^221^ Zinc–curcumin nanoparticles are free of the aforementioned drawbacks. Zinc ions enhance the solubility of curcumin and increases it cellular uptake. Zinc nanoparticles release drug in tumor sites in response to pH. Because of its high cellular uptake, siRNA–elF5A2 enters readily into cancer cells. Co-delivery of curcumin and siRNA–elF5A2 inhibits proliferation and malignancy of bladder cancer cells both in vitro and in vivo. The combination induces apoptosis of the bladder cancer cells via upregulation of Bax and down-regulation of Bcl-2 (Figures 2 and 4).^222^

One of the most well-known phytochemicals in anticancer therapy is curcumin. Many cell culture and animal experiments have been performed to evaluate its antitumor activity against different types of cancer. The poor bioavailability of curcumin may be resolved by coadministration with piperine derived from black pepper or using nanoparticles that significantly promote curcumin accumulation in cancer cells.^223^

Antitumor activity of curcumin may be improved by its coapplication with siRNA. For instance, siRNAs can down-regulate expression of Bcl-2, STAT3, and elF5A2 to interfere with cancer cell proliferation. This paves the way for enhanced antitumor activity of curcumin against cancer cells. A combination of curcumin and siRNA, and their co-delivery by nanoparticles can provide effective anticancer therapy. To date, only a few studies have evaluated the efficiency of this combination. Further studies should focus on the ability of curcumin and siRNA in down-regulation of other signaling networks, such as Nrf2, Wnt, c-Myc, and SOX in anticancer therapy. Other nanocarriers, such as micelles, liposomes, and carbon nanotubes can be designed for co-delivery of curcumin and siRNA. Table 3 represents curcumin–siRNA co-delivery in anticancer therapy.

**Table 3:** Curcumin–siRNA Co-delivery in Anticancer Therapy

nanovehicle	cancer type	cell line	target gene	size (nm)	zeta potential (mV)	encapsulation efficiency (EE) (%)	remarks	refs
PAMAM dendrimer	liver cancer	HeLa cells	Bcl-2	180	–48	82	high cellular uptake, synergistic impact, down-regulation of Bcl-2 and stimulation of apoptosis	(211)
cationic liposome	skin cancer	mouse melanoma cells (B16F10)	STAT3	276.9	42.8	86.8	down-regulation of STAT3 and effective inhibition of tumor growth and viability	(216)
cationic liposome	skin cancer	human epidermoid carcinoma cells (A431)	STAT3	195	58.8	87.5	significant reduction in STAT3 expression, resulting in inhibition of cancer growth and invasion	(217)
Zn nanoparticle	bladder cancer	human bladder cancer cell line	elF5A2	80–500	+22.3		effective knock-down of elF5A2, induction and apoptosis and reducing proliferation and growth of cancer cells	(222)

### Taxane–siRNA Co-delivery

#### Docetaxel–siRNA Co-delivery

Docetaxel (DTX) is a semisynthetic taxane derived from the needles of the European yew tree.^224^ This chemotherapeutic agent functions by inhibiting cell replication via interfering with microtubule network and stimulation of cell cycle arrest.^225^ The US FDA has approved the application of docetaxel for the treatment of lung cancer,^226^ prostate cancer,^227^ ovarian cancer,^228^ and breast cancer.^229^ Several clinical trials have evaluated the efficacy of docetaxel in anticancer therapy, and it is considered as an ideal candidate in chemotherapy of cancer patients.^230−232^ Different pathways and mechanisms contribute to the resistance of cancer cells in docetaxel chemotherapy. Regulation of these molecular pathways and mechanisms is important in the reversal of docetaxel resistance. Modulation of miR expression, Nrf2, and Klotho demonstrated promising results in inhibition of docetaxel resistance.^230,233,234^ More importantly, genes may be modulated by siRNA to improve the antitumor activity of docetaxel. Knockout of the oncogenes Notch1 and CIP2A by siRNA enhances the efficacy of etoposide in eradication of cancer cells.^235,236^ The antitumor activity of etoposide and potential of siRNA in gene silencing may be promoted using nanoplatforms. To date, different studies have evaluated the efficacy of co-delivery of docetaxel and siRNA using nanoparticles.

The ERK-1 (p42-MAPK) and ERK-2 (p44-MAPK) kinases can be induced by growth factors through Ras-Raf-dependent pathways and are upregulated in prostate cancer cells.^237^ Suppressing the expression of these MAPL kinases for elimination of prostate cancer cells.^238,239^ To optimize therapy against prostate cancer, a combination of etoposide and siRNA-MAPK has been experimental codelivered by polymeric nanoparticles into cancer cells. The codelivered siRNA-MAPK diminished the expression of ERK-1 and ERK-2 and suppressed the proliferation and invasion of prostate cancer cells, while the codelivered etoposide induced apoptosis and cell cycle arrest via down-regulation of α-tubulin.^240^

**Figure 5:**
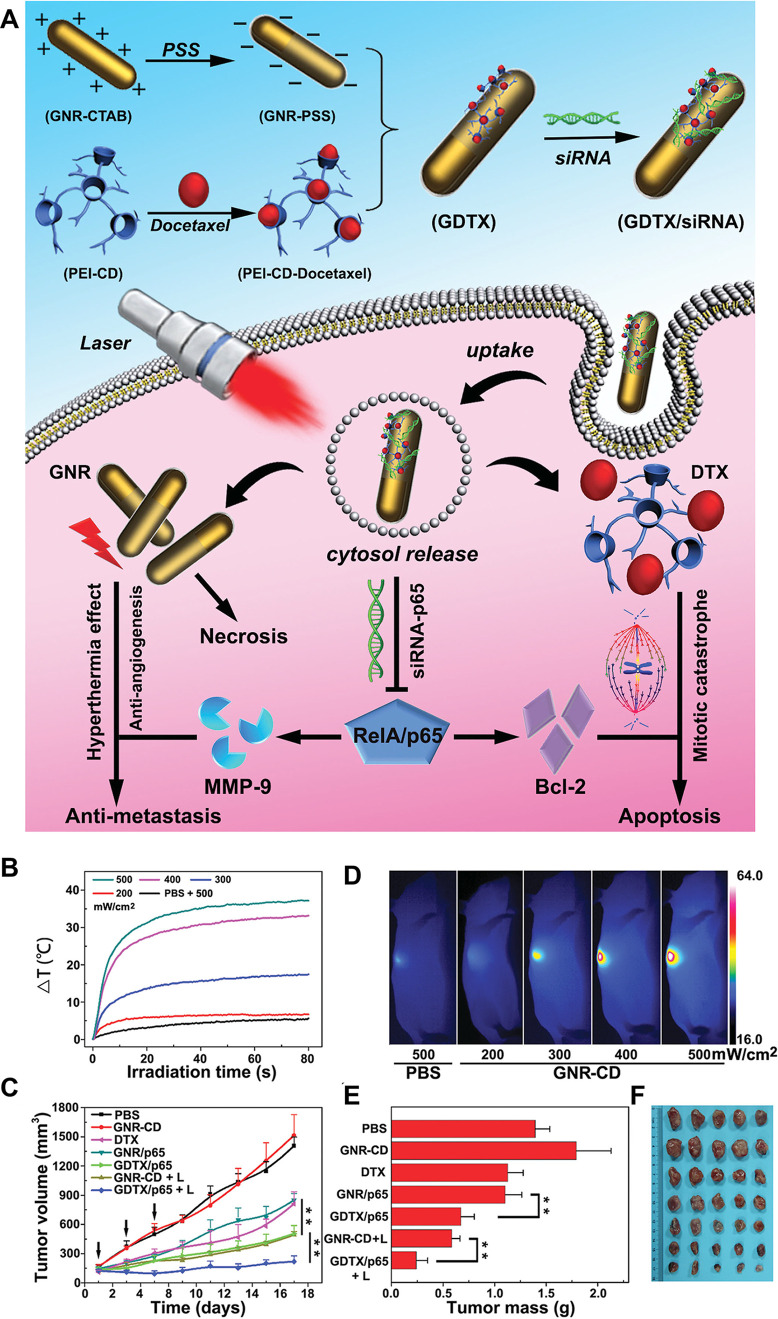
(A) Schematic illustration of the fabrication and therapeutic mechanism of siRNA and DTX coloaded host–guest gold nanorods (GNRs). (B) Temperature elevation, (C) infrared thermal images of 4T1 tumors upon laser irradiation at various power densities (200, 300, 400, and 500 mW cm ^–2^), (D) tumor growth curves, and (E) change of tumor weight after treated with GDTX/siRNA nanoparticles and 655 nm laser; the black arrows indicated the time points for DTX/siRNA injection and laser irradiation. (F) Tumor photographs with different treatment. Reproduced from ref (248) with permission from Wiley.

Matrix metalloproteinase-9 (MMP-9) is involved in the metastasis of cancer cells. This protease degrades the cell membrane of cancer cells and enhances their mobility and progression, resulting in poor prognosis.^241,242^ Because MMP-9 increases the resistance of cancer cells to chemotherapy,^243,244^ it is a suitable target in anticancer therapy. A potential strategy combining docetaxel and siRNA–MMP-9 has been used experimentally for the treatment of breast cancer. The docetaxel- and siRNA–MMP-9-loaded polymeric nanoparticles inhibit migration and viability of breast cancer cells by down-regulation of MMP-9 (inhibition of metastasis) and cellular uptake of docetaxel (apoptosis induction).^245^ Because MMP-9 induces epithelial-to-mesenchymal transition via extracellular matrix degradation,^246^ it is rational to down-regulate MMP-9 to control malignancy and sensitize the cancer cells to chemotherapy.^247^ Breast cancer cells have been found in the lung due to metastasis. Down-regulation of MMP-9 enhances the overall survival of patients with breast cancer. Loading docetaxel and siRNA–p65 into nanoparticles significantly suppresses lung metastasis of breast cancer cells via inhibition of MMP-2 and Bcl-2, and stimulation of apoptosis (Figure 5).^248^ These studies indicate that nanoplatforms are beneficial in co-delivery of etoposide and siRNAs to enhance internalization of etoposide by promoting its antitumor activity and suppressing the migration and proliferation of cancer cells.^249^

Autophagy is a type II programmed cell death and plays a pivotal role in the degradation of proteins and organelles, such as the Golgi apparatus, mitochondria, and endoplasmic reticulum.^250^ Autophagy is correlated with metabolic stress, genomic damage and tumorigenesis.^251^ Autophagy is not only involved in survival and progression of cancer cells, but can increase the resistance of cancer cells to chemotherapy.^252,253^ For example, autophagy increases the resistance of cancer cells to docetaxel chemotherapy.^254,255^ Consequently, regulation of autophagy is important in cancer therapy. A combination of docetaxel and siRNA–ATG7 has been used experimentally for the treatment of breast cancer. ATG7 is an upstream inducer of autophagy.^256^ Administration of docetaxel stimulates autophagy and suppresses the proliferation and migration of breast cancer cells. Co-delivery of siRNA-ATG7 and docetaxel using micelles suppresses prosurvival autophagy in breast cancer cells and improves the efficacy of docetaxel in the stimulation of apoptosis.^257^

**Figure 6:**
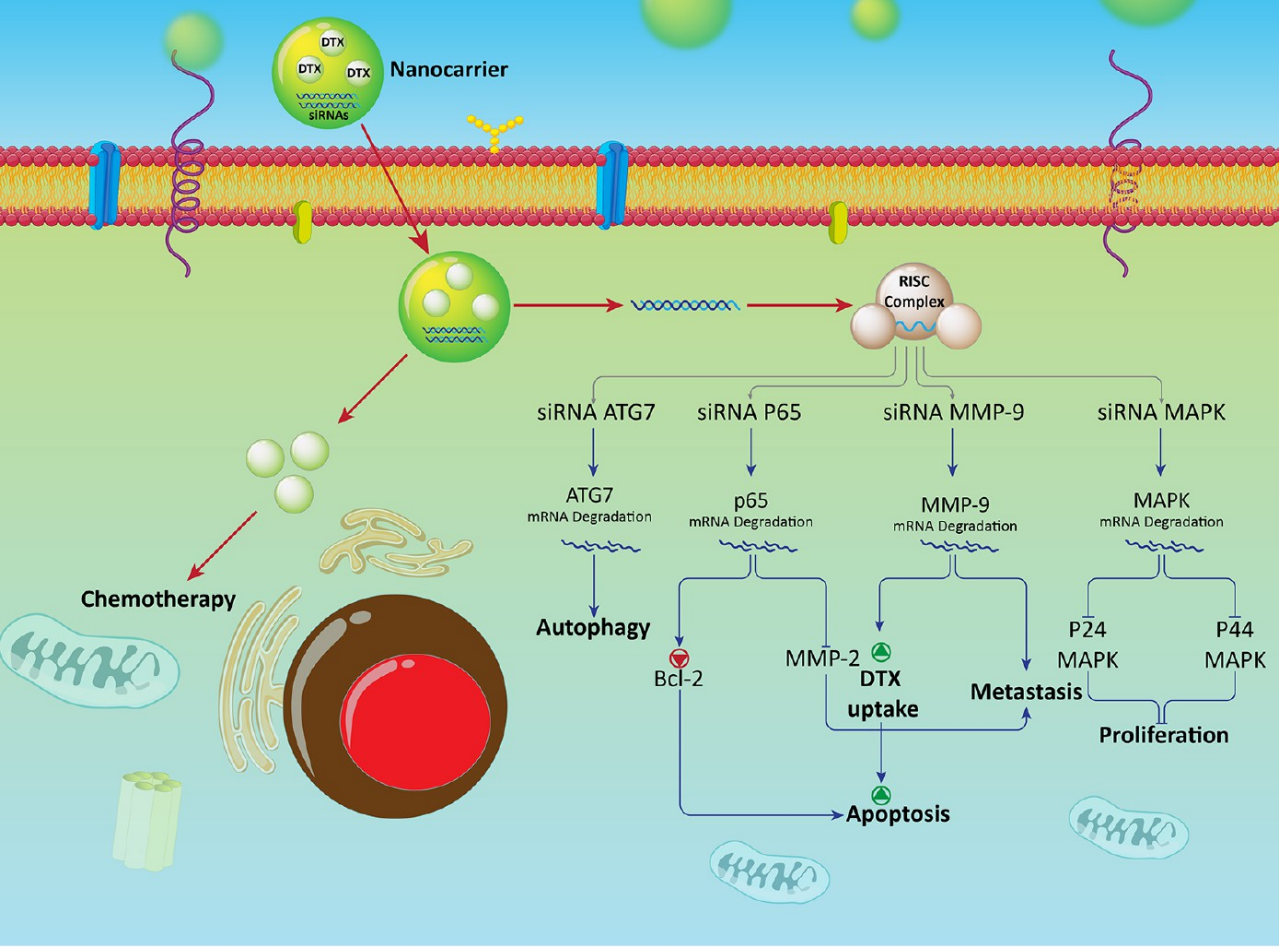
Co-delivery of docetaxel–siRNA in treatment of cancer. Suppression of the proliferation and metastasis of cancer cells is provided using siRNA-ATG7, p65, MMP-9, and MAPK. This results in increase in cytotoxicity of DTX docetaxel against cancer cells. Nanoparticles provide a platform for co-delivery of docetaxel and siRNA in triggering chemosensitivity.

The surface of nanoparticles may be modified with receptors to enhance the cellular uptake of siRNA- and docetaxel-loaded nanoparticles. The low-density lipoprotein receptor-related protein (LRP) receptor undergoes up-regulation in BBB and glioblastoma cells.^258−260^ Angiopep-2 and tLyp-1 are ligands that on bind to receptors on cancer cells and penetrate these cells.^261−264^ Surface modification of liposomes with Angiopep-2 and tLyp-1 has been performed to enhance their penetration into glioblastoma cells, resulting to increase in the internalization of docetaxel and siRNA–VEGF.^265^ Liposomes provide an effective platform for coloading of siRNA and docetaxel. This co-delivery remarkably reduced the proliferation and viability of cancer cells via induction of apoptosis.^266^ Micelles are another potential candidate for drug delivery. They are capable of encapsulating chemotherapeutic agents to improve their antitumor activity.^267,268^ The antitumor activity of siRNA–Bcl-2- and docetaxel-loaded micelles against breast cancer cells has been investigated in a recent study. The micelles codelivered siRNA and docetaxel to the tumor site. This targeted delivery significantly reduced the growth of cancer cells via induction of apoptosis and down-regulation of the antiapoptotic factor Bcl-2 (Figure 6).^269^

Similar to other antitumor agents, siRNA and nanoparticles have been successful in promoting the inhibitory effect of docetaxel against cancer cells. Proliferation (MAPK) and metastasis (MMP-9) have been down-regulated by siRNA in promoting antitumor activity of docetaxel. Nanocarriers such as polymeric nanoparticles and micelles have been used for siRNA and docetaxel co-delivery. Autophagy induction following docetaxel chemotherapy functions as a pro-survival factor. SiRNA–ATG7 inhibits autophagy in promoting the antitumor activity of docetaxel against cancer cells. Nanoparticles are potentially useful in anticancer therapy because they are capable of inducing autophagy^270−273^ and that autophagy has both oncogene and onco-suppressor functions.^274−277^Table 4 summarizes currently published docetaxel–siRNA co-delivery platforms in anticancer therapy.

**Table 4:** Docetaxel–siRNA Co-delivery Platforms in Anticancer Therapy

nanovehicle	cancer type	cell line	target gene	size (nm)	zeta potential (mV)	encapsulation efficiency (EE) (%)	remarks	ref
micelle	prostate cancer	PCa cells	SREBP1	100	+20.3 to +26.9		high cellular uptake via lysosome escape, and suppressing invasion, metastasis and proliferation of cancer cells	(278)
polymeric NPs	prostate cancer	PC-3 cell line	GRP78	39.7	–24.2	83.8 (DTX)	targeted delivery using RGD segment, high biocompatibility, excellent EE, prolonged-release and high antitumor activity	(279)
						82.4 (siRNA)		
chitosan NPs	breast cancer	Mucin1+ SKBR3 and mucin1– CHO cells	cMET	110.5	+11.6	90.7 (siRNA)	high cellular uptake, effective down-regulation of cMET, suppressing the expression of STAT3, IL-8, MMP-2, MMP-9, and VEGF, leading to a decrease in invasion and proliferation of cancer cells	(280)
						88.3 (DTX)		
chitosan NPs	breast cancer	SKBR3 breast cancer cells	IGF-1R	110–118	+12 to +14	91.2 (siRNA)	high cellular uptake, reducing cancer viability, and down-regulation of IGF-1R, STAT3, MMP-9 and VEGF	(281)
						87.6 (DTX)		
liposome	laryngeal cancer	Hep-2 cells	ABCG2	180			inhibiting tumor growth for in vitro and in vivo	(282)
polymeric NPs	nasopharyngeal carcinoma	HEN-1 cells	MMP-9				down-regulation of MMP-9, stimulation of apoptosis and suppressing metastasis	(283)

#### Paclitaxel–siRNA Co-delivery

Paclitaxel (PTX) is the first member of the taxane family that was approved by the FDA for use in clinical trials.^284^ This chemotherapeutic agent is exclusively applied in the treatment of malignancies such as breast cancer,^285^ lung cancer,^286^ brain tumors,^287^ ovarian cancer,^288^ and cervical cancer.^289^ Nevertheless, the resistance of cancer cells to paclitaxel has resulted in unfavorable outcomes in its clinical applications.^290^ Different factors are responsible for the resistance of cancer cells to paclitaxel chemotherapy, including drug transporters and miRs.^291^ Identification of these pathways and mechanisms, as well as further targeting, are beneficial for the reversal of paclitaxel resistance.^292^ For the treatment of lung cancer, siRNA–Beclin inhibits prosurvival autophagy in lung cancer cells and sensitizes the cells to paclitaxel chemotherapy. By down-regulating Beclin/autophagy, the expression and activities of P-gp and multidrug resistance protein 7 (ABCC10) are reduced. This generates the conditions for enhanced intracellular accumulation of paclitaxel to promote its potent antitumor activity.^293^ Using a combination of siRNA–VEGF and paclitaxel is also beneficial in anticancer therapy. The siRNA–VEGF suppresses metastasis of cancer cells, as well as angiogenesis and neovascularization of cancerous tissues, while paclitaxel exerts its inhibitory effect on the growth and viability of cancer cells.^294^

Stathmin 1 (STMN1) is an oncogene that promotes growth and differentiation of cancer cells.^295^ Targeting STMN1 is important in anticancer therapy. siRNA-mediated STMN1 down-regulation is correlated with enhanced sensitivity of cancer cells to paclitaxel chemotherapy.^296^ These studies support the use of paclitaxel and siRNA to promote the antitumor activity of paclitaxel and to inhibit the resistance of cancer cells to paclitaxel chemotherapy.^297^ Future research in improving the antitumor activity of paclitaxel and siRNA should be directed at the use of nanotechnology. Nanoplatforms can effectively encapsulate siRNA and paclitaxel, protecting them against degradation and providing targeted delivery to the tumorous sites.^298^ Studies that evaluated the efficacy of nanoparticles in co-delivery of siRNA and paclitaxel will be reviewed below.

**Figure 7:**
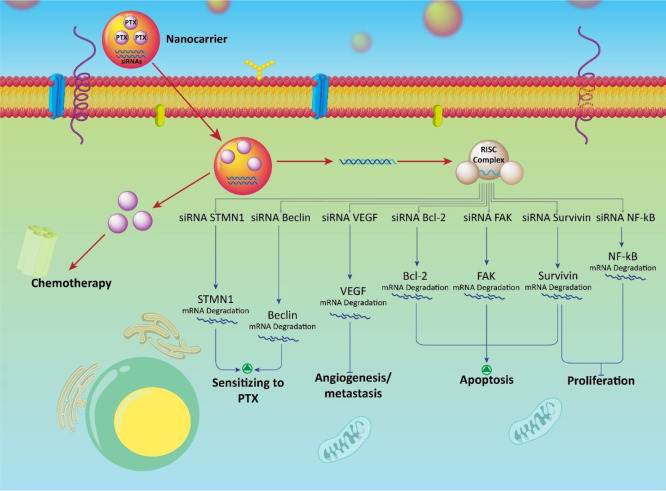
Targeting molecular pathways in anticancer therapy using paclitaxel–siRNA-loaded nanoparticles. SiRNA–Beclin inhibits autophagy and enhances the antitumor activity of paclitaxel. SiRNA–STMN1, VEGF, Bcl-2, FAK, survivin, and NF-κB sensitize cancer cells to paclitaxel chemotherapy. The potential of siRNA and paclitaxel in anticancer therapy is boosted when they are loaded into nanoparticles.

Solid lipid nanoparticles are potential nanocarriers containing physiological and biocompatible lipids. These nanocarriers have a size of 10–1000 nm and are capable of encapsulating both hydrophilic and hydrophobic drugs.^299,300^ Biocompatibility, sustained release, and biodegradability are additional beneficial characteristics of solid lipid nanoparticles.^301^ Co-delivery of siRNA–Bcl-2 and paclitaxel has been used in experimental therapy against cervical cancer. These nanocarriers induced apoptosis in cancer cells and reduced their viability and proliferation via down-regulation of Bcl-2 and stimulation of paclitaxel-mediated apoptosis.^302^ Gold nanoparticles may also be used for the delivery of siRNA because of their adjustable physicochemical features.^303^ Bifunctional polyethylene glycol moieties on the gold nanoparticles enhance targeted delivery and cellular internalization.^304^ These nanocarriers are used for co-delivery of siRNA–NF-κB and paclitaxel. Surface modification of gold nanoparticles with anisamide enhances their cellular uptake by prostate cancer cells. Anisamide acts as a ligand for up-regulation of sigma receptors in prostate cancer cells.^305,306^ Co-delivery of siRNA–NF-κB and paclitaxel via anisamide-modified gold nanoparticles effectively down-regulate NF-κB and enhanced intracellular accumulation of paclitaxel in prostate cancer cells. This resulted in halting the proliferation and invasion of cancer cells.^307^ Similar to docetaxel, there have been intense interest in targeting genes involved in the viability and survival of cancer cells, to render the cells more conducive to paclitaxel chemotherapy. For example, siRNA–survivin and paclitaxel have been loaded into cationic liposomes for antiglioma therapy. Surface modification of these liposomes by CD133 enhances their cellular uptake by cancer cells. Down-regulation of survivin, induction of apoptosis, and inhibition of proliferation result from the use of these cationic liposomes.^308^ Apart from inhibiting the proliferation and growth of cancer cells, regulating the migration of cancer cells is also of interest in anticancer therapy. This is because cancer cells with high motility result in poor prognosis.^309,310^ Inhibition of cancer cell metastasis controlling factors involved in angiogenesis. The co-delivery of siRNA–VEGF and paclitaxel by micelles suppressed the proliferation and invasion (siRNA–VEGF) of cancer cells, improving the overall prognosis (Figure 7).^311^

Focal adhesion kinase (FAK) is a novel target in anticancer therapy because its expression is up-regulated in different cancers.^312^ Overexpression of FAK increases the resistance of cancer cells to chemotherapy. Accordingly, modulation of FAK expression can provide new therapeutic venues in inhibiting chemoresistance.^313^ Surface modification of nanoparticles with hyaluronic acid (HA) enhances their penetration into cancer cells because HA binds to CD44, which is highly expressed on cancer cells.^314,315^ The HA-modified poly(lactic-*co*-glycolic acid) nanoparticles are able to target ovarian cancer cells, and have high cellular uptake because they target CD44 receptors. The siRNA–FAK reduces the resistance of ovarian cancer cells to chemotherapy and paclitaxel induces apoptosis in cancer cells.^316^ Efflux transporters and Bcl-2 are the most common targets used to render cancerous cells more susceptible to paclitaxel chemotherapy. Efflux transports such as P-gp inhibit intracellular accumulation of chemotherapeutic agents while Bcl-2 suppresses apoptosis, thereby increasing the viability and survival of cancer cells.^317,318^ Co-delivery of siRNA–Bcl-2, siRNA–MDR1, and paclitaxel via poly(lactic-*co*-glycolic acid) nanoparticles is associated with improvement in the antitumor activity of paclitaxel, inhibition of growth and proliferation of cancer cells, and increased accumulation of paclitaxel within cancer cells.^319^ Paclitaxel resistance is gradually becoming an increasing challenge in anticancer therapeutics. Overcoming paclitaxel resistance requires designing a collaborative antitumor therapy in which siRNA inhibits expression of genes involved in paclitaxel resistance. Other hurdles include eliminating the poor bioavailability of paclitaxel and enhancing its targeted delivery. Nanoplatforms are able to release paclitaxel at the tumor site and enhance its internalization.^320−330^ Co-delivery of paclitaxel and siRNA has been extensively investigated in anticancer therapy. Overall, proliferation and metastasis are negatively affected by paclitaxel and siRNA. Paclitaxel and siRNA impede angiogenesis via VEGF down-regulation to disrupt cancer metastasis. Nanoparticles are used to promote siRNA in gene silencing and paclitaxel internalization into cancer cells. Table 5 is a summary of currently reported paclitaxel–siRNA co-delivery platforms in anticancer therapy.

**Table 5:** PTX–siRNA Co-delivery Platforms in Cancer Therapy^a^

nanovehicle	cancer type	cell line	target gene	size (nm)	zeta potential (mV)	encapsulation efficiency (EE) (%)	remarks	ref
solid lipid NPs	cervical cancer	HeLa cells	Bcl-2	180	+22.2 to +48.16	97–98	down-regulation of Bcl-2, and induction of apoptosis	(331)
liposome	melanoma	B16F10 cells	Bcl-2	136	34.5	94 (siRNA)	down-regulation of Bcl-2, and inhibition of growth and proliferation	(332)
						91.2 (PTX)		
lipid NPs	breast cancer	human triple-negative breast cancer MDA-MB-231 cells	elF4E		10–60		reversal of PTX resistance and induction of apoptosis	(333)
polymeric NPs	cervical cancer	HeLa cells	E7	100–1000	–14.4 to −30	88.4 (siRNA)	effective delivery into cancer cells, enhanced accumulation of siRNA and PTX in cancer cells, down-regulation of E7 and suppressing cancer proliferation and malignancy	(334)
						90.2 (PTX)		
liposome	ovarian cancer	HeyA8-MDR cells	KSP	150.7	12.1		high cellular uptake, down-regulation of KSP, and more inhibitory effect on cancer cells compared to PTX alone	(335)
micelle	breast cancer	MCF-7	MDR1	171.6	–22.52	93.92	protection of siRNA against degradation by macrophages, down-regulation of MDR1 and suppressing tumor volume	(336)
micelle	breast cancer	MDA-MB-231 cells	AURKA	135	+14	86	delivering cargo in an HA-receptor mediated endocytosis, and high antitumor activity	(337)
polymeric NPs	breast cancer	mouse breast cancer cell lines 4T1	twist	80–140	+16 to +36	92.79	suppressing metastasis of cancer cells via down-regulation of twist	(337)
polymeric NPs	ovarian cancer	MDR ovarian cancer cell lines SKOV3TR	MDR1	173.3	–22.5		inhibiting expressions and activities of P-gp and MDR1, and suppressing PTX resistance	(338)
micelle	ovarian cancer	human ovarian adenocarcinoma resistant cell line, SKOV3-tr PXL resistant cells	survivin	25		50 (siRNA)	down-regulation of survivin, and exerting antitumor activity	(339)
						90 (PTX)		
micelle	liver cancer	human hepatocellular carcinoma (HCC) HepG2 cell	Bcl-2	394.3–427	+22		high cellular uptake, exerting antitumor activity and inhibition of Bcl-2 expression	(340)
polymeric NPs	breast cancer	human breast cancer MCF-7 cells	VEGF	120.48	+47.60		suppressing tumor growth for in vitro and in vivo	(341)

a NP: Nanoparticles.

#### Etoposide–siRNA Co-delivery

Etoposide is a member of epipodophyllotoxins that are capable of suppressing the activity of DNA topoisomerase II.^342^ This chemotherapeutic agent exerts its antitumor activity by inhibition of DNA topoisomerase and subsequent induction of DNA damage and apoptotic cell death.^343,344^ To date, etoposide has been applied in the treatment of different cancers with excellent results achieved in clinical trials.^345−347^ There is still a long way in improving the antitumor activity of etoposide. Similar to other chemotherapeutic agents, cancer cells are capable of acquiring resistance to etoposide chemotherapy.^348,349^ Studies have looked at the use of combined etoposide and gene therapy in the treatment of cancer. This regime demonstrated satisfactory results in cancer therapy. The ABCB1 is a drug transporter involved in imparting cancer cells with resistance to chemotherapy. This is achieved by controlling efflux of chemotherapeutic agents and reducing their accumulation in cancer cells that results in chemoresistance.^350^ The siRNA–ABCB1 effectively suppresses this transporter and enhances etoposide accumulation in cancer cells, thereby decreasing the viability and proliferation of cancer cells.^351^ In addition to transporters, genes participating in the survival of cancer cells may also be targeted. Silencing survivin gene using siRNA remarkably decreases the viability of leukemia cancer cells and induces their apoptosis.^352^ Another apoptotic factor is p53. The oncoprotein inhibitory member of the ASPP family (iASPP) functions as an upstream modulator of p53; iASPP reduces the expression of p53 and renders cancer cells resistant to apoptosis.^353,354^ Knock-down of iASPP by siRNA stimulates the expression of p53 and make cancer cells susceptible to etoposide-mediated apoptosis.^355^ Although the combination of etoposide and siRNA is beneficial in cancer elimination,^356^ further progress has to be made to enhance the efficacy of these agents. This may be achieved by using nanotechnology as platforms for targeted delivery of etoposide and siRNA.

Small interfering RNA may be used to knockout the genes involved in malignancy. Vascular endothelial growth factor (VEGF) is an oncogene involved in enhancing tumor neovascularization and is up-regulated in different types of cancer.^357,358^ Because of the role of VEGF in promoting cancer growth and viability, studies have been performed on the inhibition of VEGF expression in anticancer therapy.^359,360^ The combination of siRNA–VEGF and etoposide appears to be beneficial in the treatment of lung cancer. Multifunctional nanoparticles have been used as platforms for coloading of siRNA–VEGF and etoposide. The multifunctional nanoparticles are capable of codelivering siRNA–VEGF and etoposide to tumor cells because of their excellent internalization potential. The mild acidic pH of the tumor microenvironment induces the release of siRNA–VEGF and etoposide, providing targeted delivery. Effective co-delivery of siRNA–VEGF and etoposide resulted in suppression of angiogenesis and metastasis of lung cancer cells.^361^ Another oncogene in lung cancer cells is the enhancer of zeste homologue 2 (EZH2) belonging to the family of the Polycomb Group (PcG) gene. This protein is overexpressed in lung cancer,^362^ breast cancer,^363^ thyroid cancer,^364^ as well as in brain tumors.^365^ Co-delivery of siRNA–EZH2 and etoposide using multifunctional nanoparticles has been experimental used for fighting lung cancer. In vitro and in vivo experiments demonstrated that the multifunctional nanoparticles provide targeted co-delivery of siRNA–EZH2 and etoposide, decreasing the proliferation, and metastasis of lung cancer cells.^366^

**Figure 8:**
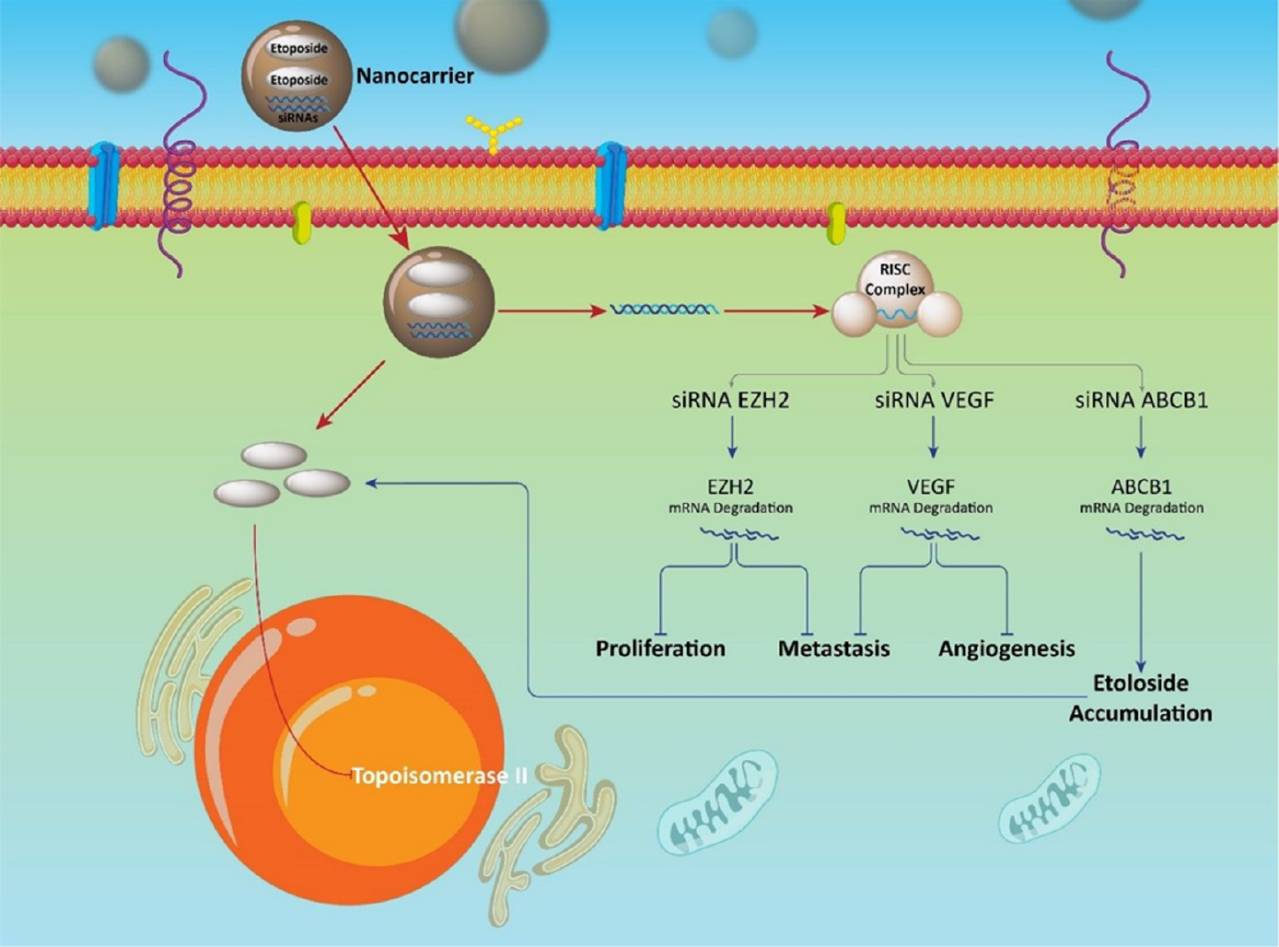
Down-stream targets of etoposide–siRNA nanoparticles in anticancer therapy. Promotion of etoposide accumulation by nanoparticles and down-regulation of ABCB1 by siRNA. This demonstrates how a combination of nanoparticles and siRNA promotes internalization of etoposide into cancerous cells. Metastasis, angiogenesis, and proliferation are suppressed following co-delivery of siRNA and etoposide by nanoparticles.

Because etoposide is frequently used for anticancer therapy, cancer cells may develop resistance to this chemotherapeutic agent. There is a need to identify the molecular pathways involved in the development of etoposide resistance. This will facilitate the design of relevant siRNA and selection of appropriate nanoparticles for targeted co-delivery of etoposide and siRNA (Figure 8).^367^Table 6 represents etoposide–siRNA co-delivery platforms in cancer therapy.

**Table 6:** Etoposide–siRNA Co-delivery Platforms in Cancer Therapy

nanovehicle	cancer type	cell line	target gene	size (nm)	zeta potential (mV)	encapsulation efficiency (EE) (%)	remarks	ref
multifunctional nanoparticles	lung cancer	A549 cells	VEGF	161.3	+15.5 to +25.5		down-regulation of VEGF, inhibition of metastasis and angiogenesis, and stimulation of apoptotic cell death	(368)
multifunctional nanoparticles	lung cancer	A549 cells	EZH2	111.7	+7.3		inhibition of EZH2, and reduction in proliferation and invasion of cancer cells	(369)

#### Resveratrol–siRNA Co-delivery

Resveratrol is a plant derived-chemical compound belonging to the flavonoid family.^372^ It has two distinct isoforms, trans-resveratrol and cis-resveratrol.^373^ These isoforms can be transformed into one another under certain circumstances. For example, exposure to ultraviolet irradiation changes the cis isoform into the trans form.^374^ Resveratrol is secreted by plants in response to biotic and abiotic stresses.^375^ This naturally occurring polyphenol provides defense against pathogens and is produced by edible plants such as hops.^376^ Resveratrol possesses excellent antioxidant, anti-inflammatory, antidiabetic, and neuroprotective activities.^377−380^ The antitumor activity of resveratrol has provided a valuable option in anticancer therapy.^381,382^ Similar to curcumin, the therapeutic effects of resveratrol are limited by its poor bioavailability.^383^ The antitumor activity of resveratrol may be accelerated by combining its use with siRNA-based gene therapy.^384^ An example if the combination of Res and siRNA–RAD51 in anticancer therapy. RAD51 is an oncogene that is involved in cancer progression and chemoresistance.^385^ Silencing of RAD51 together with the administration of resveratrol effectively induce apoptosis in cancer cells.^386^ Heat shock proteins (HSPs) are involved in malignancy and HSP27 is one of these proteins. Overexpression of HSP27 causes metastasis of cancer cells via induction of epithelial–mesenchymal transition.^387,388^ A combination of resveratrol and siRNA–HSP27 significantly inhibited the proliferation and migration of glioblastoma cells via down-regulation of HSP27 and activation of caspase-3, which, in turn, causes apoptosis of the cancer cells.^389^

**Figure 9:**
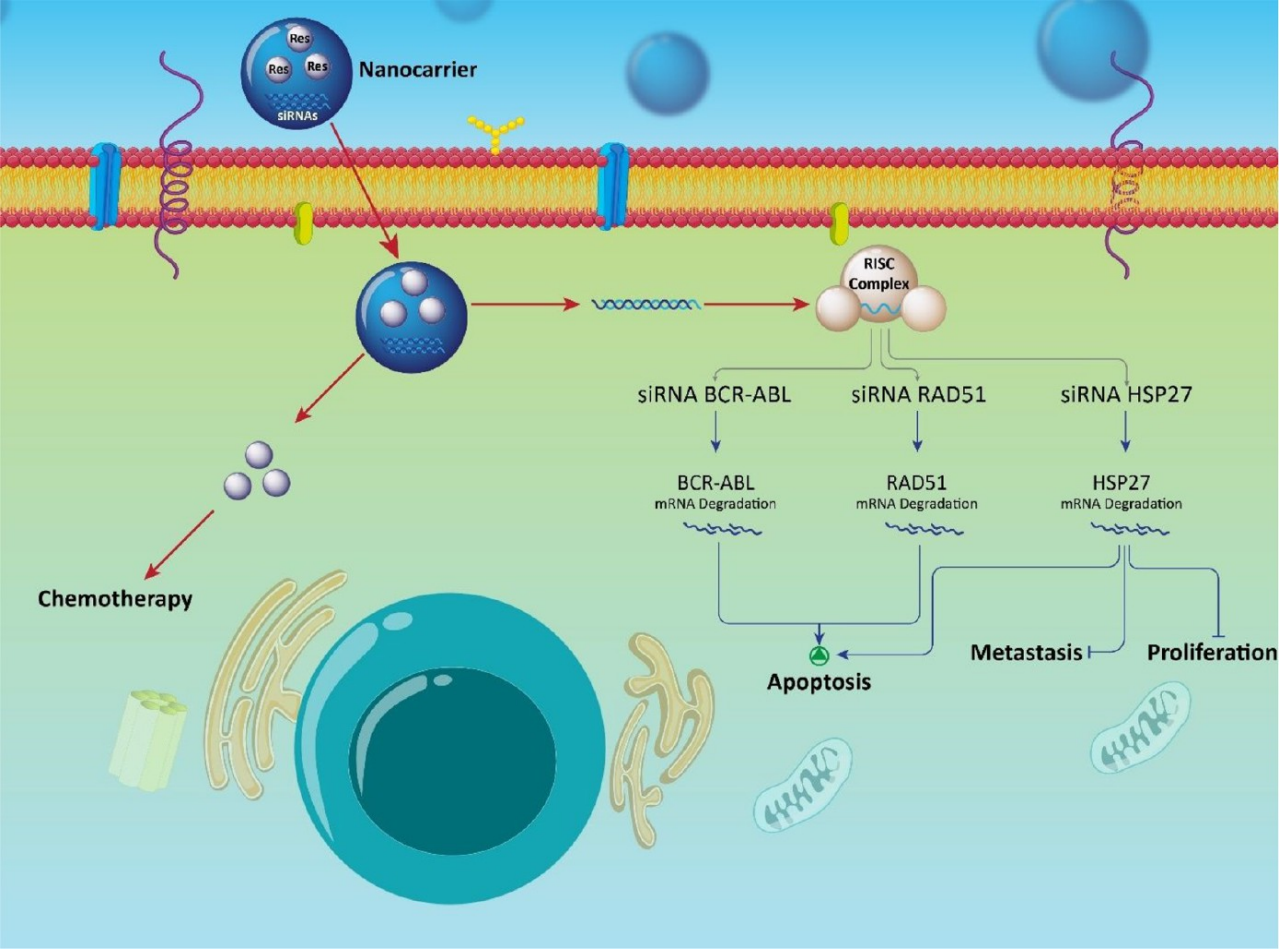
Targeting molecular signaling pathways in cancer therapy using resveratrol–siRNA-loaded nanoparticles. Apoptosis induction via siRNA–RAD51 and HSP27 results in increase in the antitumor activity of resveratrol. Co-delivery of resveratrol and siRNA by nanoparticles enhances their cellular uptake and antitumor potential.

The use of nanoplatforms for co-delivery of Res and siRNA enhances their antitumor activity. Over the past decades, electrospun fibers have been considered ideal candidates for drug delivery because of their potential in acting as platforms for sustained drug release.^390^ Multilayered core–shell fibers can be formed using multiaxial electrospinning. Drugs with different release kinetics may be incorporated in different compartments of the core–shell fibers.^391^ These electrospun fibers for delivery of resveratrol to cancer cells. Resveratrol- and siRNA-loaded electrospun fibers have been reported to reduce the viability and proliferation of leukemia cells. This is due to prolonged-release of resveratrol in 5 days and effective delivery of resveratrol and siRNA to the tumor cells.^370^ Apart from incorporating into a single nanoplatform, resveratrol and siRNA may be loaded into two distinct nanocarriers. siRNA–BCR-ABL liposomes and resveratrol-loaded electrospun fibers have been prepared to reduce the viability and growth of leukemia cancer cells via sustained drug release.^371^ To date, only two studies have investigated the co-delivery of resveratrol and siRNA in anticancer therapy. Further studies should focus on the development of other nanocarriers, such as polymeric nanoparticles, solid lipid nanoparticles, niosomes, or carbon dots for co-delivery of resveratrol and siRNA currently reported (Figure 9). Table 7 represents resveratrol–siRNA co-delivery platforms that have been used experimental in anticancer therapy.

**Table 7:** Resveratrol–siRNA Co-delivery Platforms in Cancer Therapy

nanovehicle	cancer type	cell line	target gene	size (nm)	zeta potential (mV)	encapsulation efficiency (EE) (%)	remarks	ref
electrospun fiber	leukemia	K562 cells	BCR-ABL			76.9–88.3	effective delivery of Res and siRNA, and reducing proliferation and viability of cancer cells	(370)
electrospun fiberLiposome	leukemia	K562 cells	BCR-ABL	117.2	–11	85.9	releasing Res in a prolonged-release behavior, knock-down of BCR-ABL gene and decreasing viability and proliferation of cancer cells	(371)

#### Camptothecin–siRNA Co-delivery

**Figure 10:**
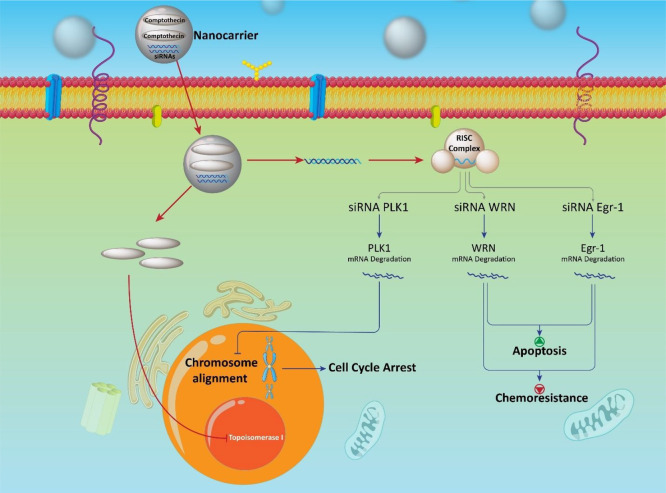
Camptothecin and its co-delivery with siRNA in the treatment of cancer.

Adverse effects and off-targeting of chemotherapeutic agents are two critical drawbacks associated with their use.^392^ Camptothecin is a potential chemotherapeutic agent capable of targeting DNA topoisomerase I by suppressing its activities in DNA transcription, replication, and chromosome condensation.^393−395^ Because the antitumor activity of camptothecin is not affected by P-gp/MDR1 resistance, it is a valuable option for anticancer therapy.^396^ Nevertheless, modifications in the administration of camptothecin should be performed to enhance its antitumor activity. Camptothecin and siRNAs (siRNA–WRN and siRNA–Egr-1) can be coadministered in anticancer therapy to induce apoptosis of cancer cells, impair their proliferation, and suppress chemoresistance.^397,398^ Nanocarriers may be used to overcome the drawbacks associated with camptothecin (side effects and off-targeting). Different nanoparticles have been used for delivery of camptothecin in anticancer therapy. Examples include polymeric nanoparticles, dendrimers, micelles, nanofibers, carbon nanotube, and multifunctional nanocarriers (Figure 10).^173,399−405^

Although camptothecin-loaded nanocarriers demonstrate potential in reducing the survival and proliferation of cancer cells, the antitumor activity of camptothecin may be further optimized via co-delivery of siRNA. Polo-like kinase 1 (PLK1) is a key member of the PLK family and has important biological functions, such as bipolar arrangement of centrosomes, spindle assembly checkpoint, and cytokinesis.^406−408^ Targeting of PLK-1 offers novel opportunities for anticancer therapy because of its roles in chromosome alignment and the cell cycle.^409−411^ The liposomes are capable of release siRNA–PLK1 and camptothecin at tumor in a sustained-release behavior. Release occurs in response to the low pH of the tumor microenvironment. The siRNA–PLK-1 and camptothecin accumulate at the tumor site, the toxicity of which causes apoptosis of the cancel cells.^412^ Apoptosis results from the inhibitory effect of camptothecin on DNA topoisomerases and silencing of PLK1. Liposomes enhance the accumulation of siRNA and camptothecin at the tumor sites. Camptothecin is the least investigated antitumor agents that we have discussed so far. Liposomes are the only nanocarriers that have been applied for co-delivery of camptothecin and siRNA against cancer. Other nanoparticles such as micelles, polymeric nanoparticles, carbon nanotubes and metal nanoparticles may also be applied for co-delivery of siRNA and camptothecin. Further studies will help in identifying the efficacy of different types of nanocarriers in promoting the antitumor activity of codelivered camptothecin and siRNA.

## Conclusion and Remarks

The efficacy of nanocarriers for the co-delivery of siRNA and natural products in the treatment of cancer was examined in the present Review. Cancer cells develop resistance against chemotherapeutic agents. Thus, ushering scientists to provide new regimes and strategies in field of anticancer therapeutics. Natural products are used in chemotherapy because of their excellent antitumor activity and their capability to target different molecular pathways. Two strategies may be considered in the investigation of the antitumor activity of natural products. The first strategy should focus on targeted delivery of chemotherapeutic agents and enhancement in their intracellular accumulation via the use of nanoparticles. The poor bioavailability of many phytochemicals may be overcome using nanoparticles. There are other barriers that limit the antitumor activity of natural products. Nanosized encapsulants derived from polymer and lipid organic nanomaterials, as well as inorganic-based nanometals, have been designed to carry siRNA and natural compounds. Encapsulants can inhibit proliferation and of cancer cells via co-delivery of natural compounds and siRNA. On one hand, this enhances the effectiveness of siRNA in gene silencing. On the other hand, the nanocarriers ameliorate the accumulation of natural products in tumor cells. As an example, in treatment of brain tumors, the BBB restricts the infiltration of antitumor agents into the brain. Nanocarriers promote penetration of the anticancer therapeutic agents through the BBB. Different receptors, such as transferrin, can be incorporated on nanoparticles for promoting their infiltration through the BBB. The BTB is another impediment that limits the penetration of antitumor agents into tumors. Nanoparticles can facilitate the penetration through BTB and promote internalization of antitumor agents. Hence, nanotechnology is an inevitable part of anticancer therapy.

Uncontrolled metastasis and proliferation of cancer cells are responsible chemoresistance. SiRNAs suppress cancer cell metastasis (MMP-9) and proliferation (Bcl-2). They improve the sensitivity of cancer cells to natural products with anticancer properties. The off-targeting limitation of siRNA may be improved via the use of nanotechnology. Nanovehicles also protect siRNA and natural compounds from degradation during blood circulation. Thus, nanocarriers, siRNA, and natural products may be combined for effective treatment against cancer. Nevertheless, these treatment regimes are still at their infancy of development. Additional animal studies are required to improve their efficacy prior to the implementation of human clinical trials.

Some studies have examined the overexpression of specific receptors on cancer cells, and have designed novel nanoencapsulant for targeting those receptors via surface modification. In addition to targeted delivery, the second strategy may be directed toward targeting molecular pathways and mechanisms involved in chemoresistance. These pathways may be utilized for increasing the sensitivity of cancer cells to chemotherapy. The siRNAs may be used for realizing the second strategy.

## Abbreviations

WHO: World Health Organization
P-gp: P-glycorprotein
FDA: Food and Drug Administration
ER: endoplasmic reticulum
ROS: reactive oxygen species
cyt C: cytochrome C
EMT: epithelial-to-mesenchymal transition
BTB: blood–tumor barrier
RNAi: RNA interference
siRNA: small interfering RNA
ssRNA: single stranded RNA
RISC: RNA-induced silencing complex
RSF-1: remodeling and spacing factor-1
GLUT-1: glucose transporter-1
MMP-2: matrix metalloproteinase-2
RR: ribonucleotide reductase
miRs: microRNAs
MDR: multidrug resistance
BBB: blood-brain barrier
DOX: doxorubicin
RAC1: Ras-related C3 botulinum toxin substrate 1
NADPH: nicotinamide adenine dinucleotide phosphate
NOX: NADPH oxidase
PD-L1: programmed death-ligand 1
CCMNPs: cancer cell membrane-coated nanoparticles
JIP1: JNK-interacting protein 1
GO: graphene oxide
PAMAM: polyamidoamine
DTX: docetaxel
MMP-9: matrix metalloproteinase-9
ECM: extracellular matrix
PCD: programmed cell death
LRP: low-density lipoprotein receptor-related protein
PTX: paclitaxel
STMN1: stathmin1
SLNs: sold lipid nanoparticles
FAK: focal adhesion kinase
HA: hyaluronic acid
iASPP: inhibitory member of ASPP family
VEGF: vascular endothelial growth factor
EZH2: enhancer of zeste homologue 2
PcG: polycomb group
Res: resveratrol
PLK: polo-like kinase
CHOP: C/EBP homologous protein
